# Healthy Eating Index-2015 Scores Vary by Types of Food Outlets in the United States

**DOI:** 10.3390/nu13082717

**Published:** 2021-08-07

**Authors:** Magdalena Vinyard, Meghan Zimmer, Kirsten A. Herrick, Mary Story, Wenyen Juan, Jill Reedy

**Affiliations:** 1 Epidemiology and Genomics Research Program, Risk Factor Assessment Branch, National Institutes of Health, National Cancer Institute, Bethesda, MD 20892, USA; magdalena.wilson@gmail.com (M.V.); meg.zimmer@nih.gov (M.Z.); kirsten.herrick@nih.gov (K.A.H.); 2 Global Health and Family Medicine and Community Health, Duke University, Durham, NC 27708, USA; mary.story@duke.edu; 3 Center for Food Safety and Applied Nutrition, Food and Drug Administration, College Park, MD 20740, USA; wenyen.juan@fda.hhs.gov

**Keywords:** HEI-2015, dietary patterns, food environment, nutrition policy, National Health and Nutrition Examination Survey (NHANES), What We Eat in America

## Abstract

Diet quality in the United States is improving over time but remains poor. Food outlets influence diet quality and represent the environments in which individuals make choices about food purchases and intake. The objective of this study was to use the Healthy Eating Index-2015 (HEI-2015) to evaluate the quality of foods consumed from the four major outlets where food is obtained—stores, full-service restaurants, quick-services restaurants, and schools—and to assess changes over time. This cross-sectional study used 24 h dietary recall data from eight cycles (2003–2004 to 2017–2018) of the National Health and Nutrition Examination Survey (NHANES). Linear trend estimation was used to test for changes in HEI scores over time, and balanced repeated replicate weighted linear regression was used to test for differences in total and component scores between types of food outlets. Overall, Americans are not consuming a mix of foods from any major category of food outlet that aligns with dietary guidelines. The total score for schools (65/100 points) and stores (62/100 points) was significantly higher than full-service (51/100 points) and quick-service (39/100 points) restaurants (*p* < 0.0001). HEI scores significantly improved over time for schools (*p* < 0.001), including an increase in whole grains from less than 1 point in 2003–2004 to 7 out of 10 points in 2017–2018. In 2017–2018, schools received the maximum score for total fruits, whole fruits, and dairy. Continued research on strategies for improving the quality of foods consumed from restaurants and stores is warranted.

## 1. Introduction

Diet quality in the United States (U.S.) is improving over time but remains poor, with a majority of Americans not meeting federal dietary guidelines [1,2]. Improving diet quality across the U.S. population can reduce the burden of chronic diseases, including obesity, diabetes, cardiovascular disease, and cancer [3,4]. Changes to the food environment have been shown to improve diet, prevent obesity, and reduce risk of chronic disease [5,6,7]. Food environment research is scalable and often designed with policy levers [8], so it is important to understand how food outlets influence diet quality at the population level.

One measure to quantify and monitor diet quality of the U.S. population is the Healthy Eating Index (HEI). The HEI measures the degree to which a set of foods aligns with the Dietary Guidelines for Americans (DGA). The DGA are updated every five years, and an updated HEI reflective of those changes is released to correspond to the new DGA. The HEI-2015 is the most recent version of the HEI, designed to reflect the 2015–2020 DGA [9,10]. The HEI is a density-based measure of diet, measuring diet quality independent of quantity, and uses a set of universal standards. These two features allow the HEI to be used to evaluate the quality of any mix of foods. When applied to different levels of the food stream, diet quality can be compared between levels and among different types of food outlets [11]. The HEI-2015 and previous versions of the HEI have been used to evaluate many levels of the food stream, such as food banks [12], food assistance programs [13,14], fast food restaurants [15,16], foods advertised in grocery circulars [17], and schools [18]. Much of this previous work has focused on the quality of foods in terms of what is offered [11,15,16,18], marketed [17,19], distributed, [12,13,14], or sold [20] in each venue. The potential variations among quality of foods actually consumed at these outlets is less understood.

The National Health and Nutrition Examination Survey (NHANES) provides a source of nationally representative data on the diet of Americans and the source of each food consumed. The NHANES data have been used to study the quality of meals consumed from food outlets such as schools and restaurants [21,22]. A recent study evaluated diet quality based on two different diet quality scores [23]. There remains a gap in the literature comparing the quality of foods consumed from different food outlets and changes over time using the most recent NHANES data and the advised analytical technique, the population ratio method, to obtain HEI-2015 scores [24]. This study fills this gap by using HEI-2015 scores to compare the quality of foods consumed by Americans from different food outlets (stores, restaurants, and schools) and assess changes over time using the population ratio method.

## 2. Materials and Methods

### 2.1. Study Design

Data on national dietary intake by food outlet are from What We Eat in America, the dietary component of the cross-sectional National Health and Nutrition Examination Survey (NHANES). The NHANES is conducted by the National Center for Health Statistics (NCHS) of the Centers for Disease Control and Prevention. The NHANES is a continuous survey conducted in two-year cycles. Due to the complex multistage probability cluster-sampling design, NHANES data are representative of the U.S. civilian population. Interviewers administer questionnaires to NHANES participants at home, followed by physical measurements and additional questionnaires in a mobile exam center (MEC), with remaining questionnaires administered over the phone [25]. The dietary data are collected via 24 h recalls (24 h) using the USDA’s Automated Multiple Pass Method [26]. Two interviewer administered 24 h recall interviews are completed: the first in-person at the NHANES mobile exam center, the second via telephone. The NCHS Research Ethics Review Board reviewed and approved all study protocols for the NHANES. Since all NHANES data were de-identified and did not contain sensitive information, this study was exempt from further review.

Eight NHANES cycles from 2003–2004 to 2017–2018 were used. The 2003–2004 NHANES cycle is the first in which a question was added to collect information on the food outlet where reported foods were obtained. The 2017–2018 cycle is the most recent cycle available. Each food item reported in the dietary intake survey was linked to the MyPyramid Equivalents Database (MPED) or Food Patterns Equivalents Database (FPED) that corresponds with each cycle of NHANES. This linkage allowed food to be disaggregated into guidance-based food groups, which were used to calculate HEI-2015 total and component scores. Both the MPED and FPED convert reported food and beverage items into 37 disaggregated USDA food pattern components (e.g., cup equivalents of fruits, ounce equivalents of whole grains, teaspoon equivalents of added sugars) [27].

### 2.2. Food Outlet Categorization

Since the 2003–2004 cycle, NHANES participants have reported the outlet where the foods consumed were obtained, selecting from multiple options. In this study, the options were collapsed into seven mutually exclusive categories:

1. Store: Grocery/supermarket, store-convenience type, store—no additional information;
2. School: Cafeteria in a K–12 school;
3. Full-service restaurant: Restaurant with waiter/waitress, bar/tavern/lounge, restaurant—no additional information;
4. Quick-service restaurant: Restaurant fast food/pizza, cafeteria not in a K–12 school, vending machine, sport/recreation/entertainment facility, street vendor/vending truck;
5. Community food program: Family/adult day care center, soup kitchen/shelter/food pantry, meals on wheels, community food program—other, community program—no additional information;
6. Homegrown: Grown by you or someone you know, fish caught by you or someone you know;
7. Other: Common coffee pot or snack tray, from someone else/gift, mail order purchase, residential dining facility, fundraiser sales, other (specify).

Frequencies were run for each NHANES cycle for all seven categories, and it was determined that community food program (n = 84–142) and homegrown (n = 234–447) did not have enough reported foods for comparison analysis. Additionally, the “other” category (n = 1905–2546) was comprised of a mix of food outlets that made data interpretation challenging. These food outlets were included in the “all outlets” category.

Retail food outlets are defined as all food sources excluding restaurants where foods are purchased by consumers then consumed off-premises. Retail food outlets include both grocery stores/supermarkets and convenience stores. Grocery stores/supermarket and convenience stores share common intervention strategies, including the four Ps of marketing: product, promotion, placement, price [28]. Grocery/supermarket and convenience stores are frequently studied together in the literature, [8] and both are included in a national research agenda for healthy food retail research [29]. Therefore, grocery store/supermarket and convenience stores were collapsed into one food retail outlet category, labeled as “stores” for the present analysis.

The final categorization of food outlets included stores, full-service restaurants, quick-service restaurants, and schools. This categorization of food outlets was based on the Scientific Report of the 2015 Dietary Guidelines Advisory Committee (DGAC), which compared the food group density over time from 2003–2004 to 2009–2010 at each of the four major types of food outlets in the U.S.: stores, full-service restaurants, quick-service restaurants, and schools [30]. This study extends the work of the 2015 DGAC in four ways: (1) updating the analysis with the most recent NHANES cycles (i.e., adding 2011–2012 to 2017–2018 cycles); (2) calculating the HEI-2015 component and total scores for each of the four major types of food outlets; (3) comparing patterns of HEI-2015 component scores between different food outlets using the population ratio method; and (4) studying changes over time.

### 2.3. Healthy Eating Index

The total HEI-2015 score and the pattern of HEI-2015 component scores were used to compare the quality and pattern of foods consumed from each of the four food outlets of interest. The HEI-2015 standards are based on the key recommendations from the 2015–2020 Dietary Guidelines for Americans (DGA), such that a total score of 100 points indicates optimal alignment with the 2015–2020 DGA [3,10]. The HEI-2015 includes thirteen components, each of which reflects an important aspect of diet quality. Nine components focus on adequacy, i.e., foods to eat enough of to get the nutrients needed for good health: total fruits, whole fruits, total vegetables, greens and beans, whole grains, dairy, total protein foods, seafood and plant proteins, fatty acid ratio. Four components focus on moderation, i.e., foods to limit or consume in small amounts: refined grains, sodium, saturated fats, added sugars. High component scores indicate that the mix of foods aligns with federal dietary guidance and low scores indicate that the mix of foods does not [10]. HEI scores were calculated from the NHANES 24 h recall using the population ratio method. Although a recent publication calculated HEI-2015 scores as the mean per-person quality of foods, prior analyses have shown that the population ratio method provides a less biased estimate of mean HEI scores for a population [31]. Thus, to calculate the mean total and component HEI scores to describe dietary intake for a population/group, the population ratio method is currently recommended [24,32]. For each of the four main food outlets, nutrients, guidance-based food groups, and calories were aggregated using a weighted sum and then translated into a density by dividing the total amount of each nutrient or food group by the total amount of calories consumed (or, in the case of the fatty acid ratio, by dividing the sum of mono- and poly-unsaturated fatty acids by the amount of saturated fatty acids). Further information on the steps for calculating HEI scores and analytic guidance including SAS code are available on the NCI webpage [33].

To visualize differences in the patterns of the thirteen component scores, the component scores were scaled as a percentage of the maximum component score and then plotted on radar plots. On the radar plots, the center point of the graph represents a score of zero, and the outer point of each axis represents the maximum score for each component. Each HEI-2015 component forms an axis on the graph, and the plot connects the scores for each axis into a figure that, by its shape, suggests a pattern. A plot with all components at the outer point of the axis, for a total score of 100, indicates optimal alignment with the 2015–2020 DGA. Therefore, a plot with many or most points closer to the outer edge represents a food pattern that is closer to meeting the recommendations of the 2015–2020 DGA than a plot with many points closer to the center of the graph. The components are displayed in the same order for each radar plot to facilitate comparisons. Additional information on HEI visualization and radar plots can be found on the NCI website and the HEI-2015 update paper [10,34]. HEI scores can be interpreted using an A–F grading scale. HEI total scores of 90–100 points, or component scores with 90–100% of the maximum component score are assigned an “A”; total scores of 80–89 or component scores with 80–89% of maximum component score are assigned a “B”; total scores of 70–79 or component scores with 70–79% of maximum component score are assigned a “C”; total scores of 60–69 or component scores with 60–69% of maximum component score are assigned a “D”; total scores of 0–59 or component scores with 50–59% of maximum component score are assigned an “F” [10]. The quality of foods consumed from stores, schools, and restaurants based on the HEI-2015 population ratio method, interpretation of these findings using the graded approach, and visualization of these findings using radar plots are all presented in this paper for the first time.

### 2.4. Demographics and Analytic Sample

NHANES participants aged 2 years and older with complete day-1 dietary recall data were included in the study. Participants with unreliable recalls and young children consuming human milk were excluded. All reported foods and beverages from these reliable day-1 recalls were included in the analytic sample. The number of food/beverage items in the final analytic sample varied by NHANES cycle. The sample of foods/beverages reported as consumed from schools ranged from 2386 items (2017–2018 cycle) to 4696 (2005–2006). The number of food/beverage items reported as consumed from stores ranged from 69,446 (2017–2018) to 99,768 (2009–2010). The number of items reported as consumed from full-service and quick-service restaurants, respectively, were 7103 (2017–2018) to 9579 (2005–2006) and 11,270 (2015–2016) to 14,907 (2005–2006).

### 2.5. Statistical Analysis

To understand the distribution of consumption (measured in calories) across food outlets and by age, the amount of food and proportion of food consumed from each food outlet was calculated. The calories consumed at each outlet was divided by the total calories across all outlets, then multiplied by 100 to yield a percentage value of calories consumed per food outlet. This distribution of the population’s total caloric intake was stratified by the following age categories: 2–5 years of age, 6–11, 12–19, 20–40, 41–50, 51–70, and over 70. Tests for statistical significance were not performed for this calculation, as it was intended to provide an overview but not a statistical comparison of the distribution of calories consumed from each food outlet. This distribution of caloric intake by food outlet was used to complement the results and further aid interpretation.

Balanced repeated replication (BRR) weights with a Fay’s coefficient of 0.3 were calculated. The BRR weights were used in tests for both differences between outlets and differences across cycles of the NHANES to account for the complex survey design of the NHANES, including differential probability of selection and non-response. The BRR calculations were run for each cycle separately to generate eight BRR standard error values. Pairwise comparison tests were used to test for significant differences in population mean HEI-2015 scores between stores, full-service restaurants, quick-service restaurants, and schools. Confidence intervals were set to 0.95 and *p* values less than 0.05 were considered statistically significant.

To test for differences in HEI-2015 total and component scores across cycles for a given food outlet (e.g., differences across cycles for stores), the outcome of interest was the presence of a linear trend over time. To test for the statistical significance, differences in HEI-2015 scores by NHANES survey cycle were explored with a BRR-weighted linear regression model. All *p* values <0.05 were considered statistically significant. All analyses were performed in SAS version 9.4 (Cary, SC, USA).

## 3. Results

### 3.1. Distribution of Consumption across Food Outlets

**Figure 1:**
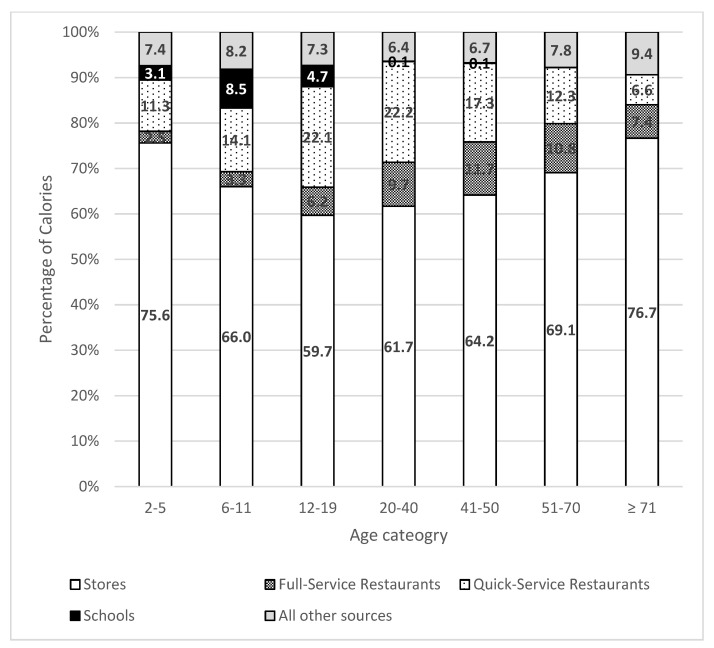
Percentage of calories obtained from each food outlet by age category, NHANES 2017–2018.

The percentage of calories that each food outlet contributed to total caloric intake by age category is presented in Figure 1, using the most recent cycle of the NHANES (2017–2018). Across all age categories, Americans consumed most of their calories from food from stores. The percentage of calories consumed from stores was lowest (60%) among those 12–19 years of age and highest (78%) among those age 71 or older. Full-service restaurants contributed 3% (2–5 years) to 12% (41–50 years) of total calorie intake, while quick-service restaurants contributed 7% (>71 years) to 22% (12–19 years and 20–40 years). The percentage of calories consumed from both types of restaurants followed a similar pattern, increasing with age, peaking in adulthood (20–40 years for quick-service, 41–50 years for full-service), then declining with age. For children and young adults <20 years, foods from schools contributed 6% of calories to the diets of children 2–5 years, 9% for 6–11 years, and 5% for 12–19 years. For the other NHANES cycles included in the study (2003–2004 to 2015–2016), similar percentages were observed (data not shown).

### 3.2. Differences in HEI-2015 Scores by Food Outlet

**Figure 2:**
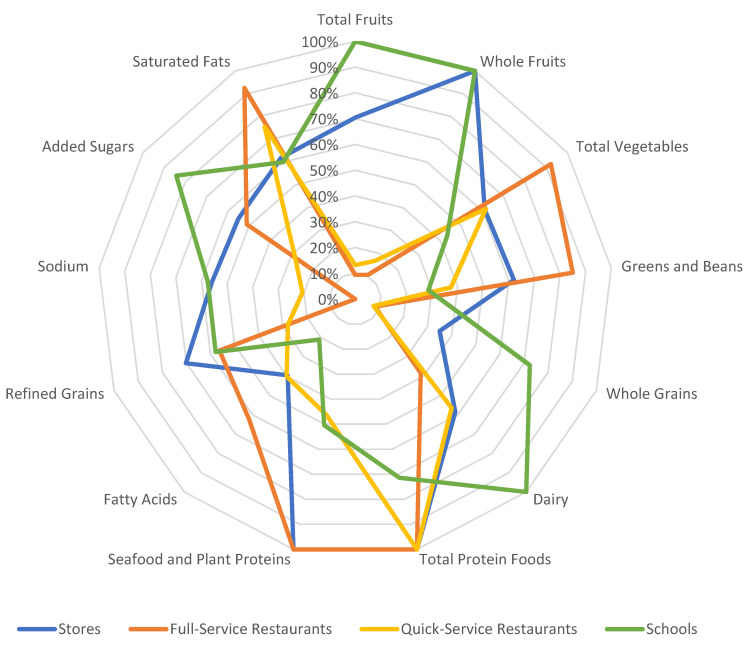
Differences in HEI-2015 component scores between types of food outlets in 2017–2018.

**Figure 3:**
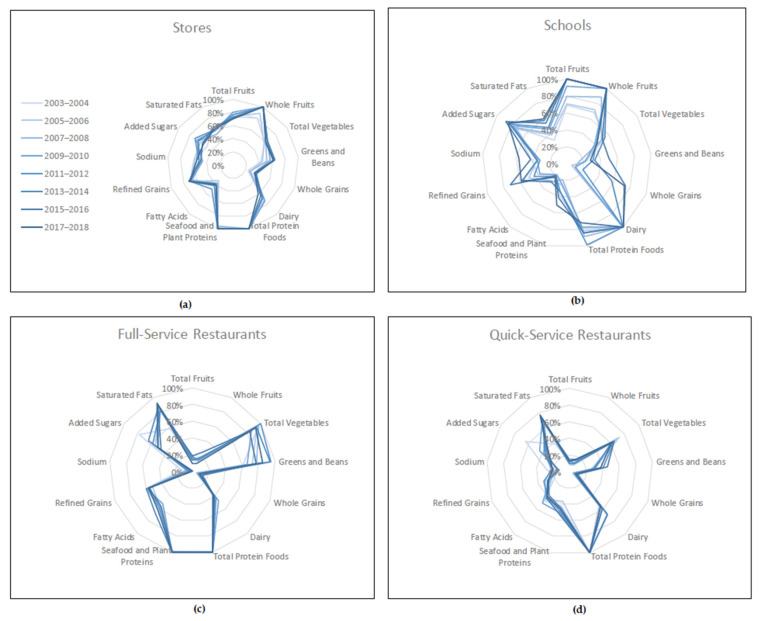
Differences in HEI-2015 component scores over time by food outlet type. (**a**) HEI-2015 component scores for stores; (**b**) HEI-2015 component scores for schools; (**c**) HEI-2015 component scores for full-service restaurants; (**d**) HEI-2015 component scores for quick-service restaurants. On the radar plots, the center point of the graph represents a score of zero, and the outer point of each axis represents the maximum score for each component. Therefore, plots with most points closer to the outer edge represent a food pattern that is closer to meeting the recommendations of the 2015–2020 DGA than a plot with many points closer to the center of the graph. Additional information on HEI visualization and radar plots can be found on the NCI website and the HEI-2015 update paper [10,34].

Overall, the total HEI scores and pattern of component scores varied substantially between food outlets. The HEI-2015 total and component scores were significantly different between outlets, with a few exceptions, as described in more detail below. Figure 2 shows a radar plot of the component scores for stores, full-service (FS) restaurants, quick-service (QS) restaurants, and schools in 2017–2018. The pattern of HEI-2015 component scores varied by food outlet, represented by the different shape per outlet. The overall patterns (shape) of component scores are much more distinct between types of outlets (Figure 2) than the changes in pattern within any one type of outlet over time (Figure 3).

On the radar plots, the center point of the graph represents a score of zero, and the outer point of each axis represents the maximum score for each component. Therefore, plots with most points closer to the outer edge represents a food pattern that is closer to meeting the recommendations of the 2015–2020 DGA than a plot with many points closer to the center of the graph. Additional information on HEI visualization and radar plots can be found on the NCI website and the HEI-2015 update paper [10,34].

In 2017–2018, schools received the maximum score for total fruits, whole fruits, and dairy, as indicated by the data points at the outermost edge of the radar plot axis. Schools scored significantly higher than stores, full-service restaurants, and quick-service restaurants for total fruits (all *p* < 0.0001), and they scored significantly higher than both types of restaurants for whole fruits (both *p* < 0.0001), but no differences were observed between schools and stores for whole fruits, as stores also received the maximum score. Schools also scored significantly higher than the three other food outlets for dairy (all *p* < 0.0001). The score for schools for whole grains was 7.24 out of 10 points, which fell short of meeting the Dietary Guidelines but was significantly higher than all three other food outlets (all *p* < 0.0001). Similarly, the added sugars score for schools was 8.43 out of 10 points, also significantly higher than the three other outlets (stores *p* < 0.0001, full-service restaurants *p* = 0.031, quick-service restaurants *p* = 0.001). The relatively high scores for whole grains and added sugars contributed to the total score of 65/100 points. Although a total score of 65 points translates to a “D” on the graded scale, it was significantly higher than the “F” received for both types of restaurants (full-service: 51 points, *p* < 0.0001; quick-service: 39 points, *p* < 0.0001). Despite scoring higher than the other types of food outlets for many component scores, schools generally scored lower for total protein foods and seafood and plant proteins, receiving scores of 3.57 and 2.52 for total protein foods and seafood and plant proteins, respectively. This total protein foods score was significantly lower than all other outlets (all *p* < 0.0001), and the seafood and plant proteins score was significantly lower than stores and full-service restaurants (both *p* < 0.0001).

Differences were also observed in the scores of foods consumed from stores vs. restaurants. In 2017–2018, stores had a higher HEI-2015 total score than both full-service and quick-service restaurants (both *p* < 0.0001) (Table S1). Additionally, stores received a significantly higher score than both types of restaurants for several components, including total fruits, whole fruits, whole grains, refined grains, sodium, and added sugars (Table 1, Figure 2). For greens and beans, stores scored significantly higher than quick-service (*p* = 0.004) but lower than full-service restaurants (*p* = 0.009). Within the restaurant category, based on the total score, both full-service restaurants and quick-service restaurants received an “F”, but the score for full-service restaurants was still significantly higher than quick-service restaurants (51 vs. 39 points, *p* < 0.0001). Full-service restaurants also scored significantly higher than quick-service restaurants for several adequacy components, including total vegetables, greens and beans, and seafood and plant proteins, (all *p* < 0.0001). Additionally, full-service restaurants scored significantly higher than quick-service for some moderation components, such as refined grains (*p* < 0.0001) (Table S1).

### 3.3. Changes to HEI-2015 Scores over Time

Overall, the HEI-2015 total score from all outlets (which included foods from the four main sources, community food programs, and homegrown foods) changed significantly over time (*p* = 0.03) (Table 1). The HEI-2015 total score for each cycle of the NHANES from 2003–2004 to 2017–2018 for each of the four food outlets of interest (stores, full-service restaurants, quick-service restaurants, schools) is displayed in Table 1. The total scores for each outlet fell below 100, indicating that the quality of foods consumed from these outlets fell short of dietary guidance. The HEI-2015 total score was highest for the mix of foods consumed from stores, with a total score between 59 (“F”) and 66 points (“D”) and has not varied significantly over time (*p* = 0.07). The total score was lowest for quick-service restaurants (38.7–41.8 points, “F”), which significantly fluctuated over time (*p* = 0.01). HEI-2015 total scores for full-service restaurants also fluctuated over time, rising and falling in the D–F range (49–54 points), but there was no significant trend (*p* = 0.34). Importantly, the total score of foods from schools significantly increased over time, from “F” (48 points) in 2003–2004 to “D” (65 points) in 2017–2018 (*p* < 0.001).

Figure 3 shows a radar plot of the patterns of HEI-2015 component scores over time from 2003–2004 to 2017–2018 for each of the four food outlets of interest: stores, full-service restaurants, quick-service restaurants, schools. Table 1 provides the detailed total and component scores for each outlet over time, as summarized in Figure 2 and Figure 3. For schools, five HEI-2015 component scores varied significantly over time: total fruits (*p* = 0.001), whole fruits (*p* = 0.01), whole grains (*p* = 0.006), added sugars (*p* = 0.002), and saturated fats (*p* < 0.001). The most significant increase was seen in the score for whole grains, which rose from less than 1 point in 2003–2004 to 7 out of 10 point in 2015–2016 and 2017–2018 (*p* = 0.006). Total fruits scores increased from 3.45 points in 2003–2004 to the maximum score of 5 points in 2011–2012, where it remained. Whole fruits followed a similar trend, beginning with 3.45 points in 2003–2004 and achieving the maximum score in 2009–2010, then staying constant at the maximum score of 5 points. The trends for added sugars and saturated fats both reflect a steady overall increase in the score (added sugars: *p* < 0.01; saturated fats: *p* < 0.001), meaning a decrease in consumption over time. Although a trend was not present for dairy, it is important to note that schools received the maximum score for dairy for all cycles of the NHANES from 2003–2004 to 2017–2018.

For stores, five component scores varied significantly over time: greens and beans, dairy, whole grains, refined grains, and added sugars (all *p* < 0.01), with the score for greens and beans generally increasing over time and scores for the other components having more fluctuation. Both full-service and quick-service restaurants had significant changes over time in the whole grain (*p* = 0.01 for full-service and *p* = 0.02 for quick-service) and added sugars (*p* = 0.02 for full-service and *p* = 0.01 for quick-service) component scores. For whole grains, the score generally increased over time among full-service restaurants but fluctuated for quick-service restaurants. For added sugars, the score fluctuated for both full-service and quick-service, though a steady decline from 2009–2010 to 2017–2018 was observed for quick-service. The scores for whole fruits (*p* = 0.02), total vegetables (*p* = 0.01), and greens and beans (*p* = 0.05) were also significant for quick-service, indicating that the pattern of food quality varied over time in more categories for quick-service restaurants than for full-service restaurants.

## 4. Discussion

This study evaluated differences in HEI-2015 total and component scores between types of food outlets and assessed changes over time. The data presented provide information on the food outlets where Americans consume a mix of foods most or least, as aligned with dietary guidelines, and which components of a healthy diet were consumed from each outlet. Overall, HEI-2015 total scores varied by food outlet and over time but remained low, with total scores at 66 points or below (i.e., at a “D” or below) for all outlets. This demonstrates that the overall quality of the mix of foods consumed from the major food outlets in the U.S. are not aligned with the 2015–2020 dietary guidelines, consistent with the 2015 DGAC report [30]. The 2015 DGAC report also found that the diet quality of the U.S. population from 2003–2010 did not meet the standards for any component of the 2010 HEI, regardless of the type of outlet where food was obtained [30]. However, this study shows that each type of food outlet received the maximum HEI-2015 component score for one or more dietary components, which may be related to improvements in scores in more recent years, i.e., from 2011–2018. The specific components receiving maximum scores, and how this changed over time, varied by food outlet. Each of the four food outlets studied—stores, full-service restaurants, quick-service restaurants, and schools—are discussed respectively in the following paragraphs.

This study found that Americans consume the greatest portion of their calories on a given day from stores, consistent with the 2015 DGAC report [30]. This is also consistent with national data on food purchases from the USDA Economic Research Service, which reports that two-thirds of calories come from grocery stores [35]. Overall, the mix of foods consumed from stores was more closely aligned with dietary guidelines than the mix of foods consumed from both full-service and quick-service restaurants, as evidenced by higher HEI-2015 total scores. This finding is consistent with knowledge that more frequent cooking at home, typically with foods obtained from stores, is associated with better diet quality and HEI-2015 scores [36]. This study found that stores consistently received the maximum score for whole fruits (starting in 2007–2008), total protein foods (all years), and seafood and plant proteins (starting in 2009–2010). Despite these positive findings, there is room for improvement, as 10 of the 13 HEI-2015 component scores fell short of dietary guidance. The influence of retail food outlets such as stores on the U.S. population’s diet quality is increasingly recognized: both the 2020–2030 Strategic Plan for NIH Nutrition Research and the 2015–2020 and 2020–2025 Dietary Guidelines for Americans recognize the important role of the food environment on dietary intake [3,37,38]. However, the food retail landscape is rapidly evolving, and knowledge gaps remain. Recently, a national research agenda for healthy food retail research was developed to build consensus in working to fill knowledge gaps [29]. Additional research aligned with this national agenda can help identify policies and corporate practices that effectively promote healthy food and beverages in stores [29]. Further, attention should be given to the contextual factors that influence intake, such as availability, affordability, and access. Access factors such as distance to a grocery store influences food outlet purchases [35].

Although stores contributed the most calories to the population’s diet, a substantial portion of calories were consumed away from home and at restaurants, with about 15–32% of calories coming from restaurants among those ages 6 to 70. The findings from this study are consistent with the literature suggesting that increasing calories consumed from food eaten away from home may not be in the best interest for the population’s diet quality. A systematic review of the literature has shown that consumption of foods away from home is linked to increased intake of energy and nutrients of public health concern [39]. Additionally, increased frequency of eating at fast food restaurants is associated with less healthful eating habits [40] and with increased risk of type 2 diabetes and cardiovascular disease mortality [41]. Unsurprisingly, this study showed that the quality of foods consumed from both full-service and quick-service restaurants earned an overall score of “F”, significantly lower than the total score for stores and schools, which both earned a “D” in 2017–2018. Quick-service restaurants in particular were the furthest from dietary guidance, with a total score of only 39 out of 100 points in 2017–2018, significantly lower than full-service restaurants. This is consistent with another study that found a larger proportion of meals at fast food restaurants to have poor diet quality, while full-service restaurants had a lower proportion of “poor” and higher proportion of “intermediate” diet quality, based on American Heart Association scores [22]. This study and previous work [22] based on consumption consider the quality of foods actually consumed from restaurants, though it can be assumed that other options, healthier or unhealthier, than those selected are offered at these locations. Nonetheless, these findings are consistent with previous work based on the quality of foods offered on fast food menus, which concluded that although diet quality of fast food menus varies, it is consistently poor across multiple fast food chains [15]. To improve diet quality, environmental and policy interventions may be among the most effective strategies for creating population-wide improvements in eating [42]. Menu labelling requirements, economic incentives such as subsidies to lower prices of healthful food items, among other evidence-based policy approaches should continue to be explored to improve diet quality in restaurant settings [43,44].

We found that the HEI total score for schools significantly and steadily increased over time. From 2015–2016 and 2017–2018, the total HEI score for schools was the highest among the four food outlets studied. The increase is likely due to the updated federal nutrition standards. The Healthy, Hunger-Free Kids Act (HHFKA) of 2010 mandated USDA to make transformative policy reforms in the school meals program, for the first time in 30 years, to improve the overall nutrition quality of school meals, which included updating the nutrition standards, which had been in place since 1995 [45]. The updated standards, consistent with the Dietary Guidelines, increased servings of fruits, vegetables, and whole grains; limited milk to fat-free and low-fat varieties; reduced the levels of sodium and saturated fats; and specified minimum and maximum levels of calories for meals. Starting in 2014–2015, all school meals were expected to meet the updated standards for breakfast and lunch. A large nationally representative study found that the nutritional quality of school meals increased significantly after the updated nutrition standards were in place [18]. For example, between 2009–2010 and 2014–2015, the total HEI-2010 score for what was offered and served in school lunches increased from 58% of the maximum score to 82% [18]. Despite this success, there have been ongoing attempts to roll back the nutrition standards, which could jeopardize the healthfulness of foods and beverages available to students [46].

Recent research has highlighted concerns of added sugars in American diets. Our study found that the added sugars score for schools was significantly higher than the other three outlets. Currently, there is no nutrition standard for added sugars in school meals. Fox and colleagues evaluated levels of added sugars in school meals [47]. The majority of schools exceeded the Dietary Guidelines limit for added sugars (no more than 10% of calories from added sugars each day) at breakfast (92%), and 69% exceeded the limit at lunch [47]. This indicates the importance of federal, state, and local policies for strengthening nutrition standards and school food policies. Further, our findings underscore the overall success and progress in schools that food and nutrition policies can make to improve population health and that could be applied to other settings; for example, scaling up effective food retail policies in restaurants and stores to the national level.

### Considerations

Individuals do not make food choices in isolation. Rather, their eating behaviors are influenced by a myriad of contextual factors, including what types of food are available to them where they live, work, and shop. The density-based scoring approach of the HEI-2015 allows it to be used to evaluate any mix of foods, [10] and it can be applied to different levels of the food stream [32]. In this study, the HEI-2015 was used to evaluate the quality of the mix of foods consumed from four food outlets. Although availability, accessibility, and affordability interact to influence consumption, variables for these constructs were not available in the NHANES dataset and therefore were not included in the present analysis.

The findings should be interpreted in the context of several other considerations. This study provides information on the quality of foods consumed from four types of food outlets using nationally representative data from a given day of intake, which provides a high degree of generalizability, though the estimates do not represent long-term or usual intake. A strength of the study was the measure of quality of foods consumed, rather than foods served or sold, some of which may be lost as waste. Since each outlet analyzed contributed a portion of the population’s total daily calorie intake (i.e., no single outlet was the sole source of calories), no food outlet was expected to achieve the maximum score of 100 points. Additionally, not all outlets where food is obtained were examined in this analysis; however, the four outlets presented represent more than 90% of all calories consumed for most cycles of NHANES analyzed. Lastly, the food environment landscape is rapidly evolving. The most current definition of retail food outlets was used to collapse supermarket/grocery stores and convenience stores into a single retail food outlet category called stores. However, future work should consider differences between large and small retail food outlets.

## 5. Conclusions

This study provides nationally representative data on the quality of foods consumed from the four major types of food outlets in the U.S. Americans are not consuming a mix of foods that aligns with the U.S. Dietary Guidelines from any of the major food outlets, and foods consumed from quick-service restaurants fall particularly short of national dietary guidance. However, some types of food outlets have demonstrated significant positive improvements in HEI-2015 scores over time, especially schools. The increase in scores for schools is consistent with the implementation of the Healthy, Hunger Free Kids Act, demonstrating a potential opportunity for policy impacts of other food outlets such as stores and restaurants on the American population’s diet quality.

## Figures and Tables

**Table 1:** HEI-2015 total and component scores by food outlet category per NHANES cycle 2003–2018.

			HEI-2015 Component Scores												
Outlet	Year	HEI-2015 Total Score	Total Fruits	Whole Fruits	Total Vegetables	Greens and Beans	Whole Grains	Dairy	Total Protein Foods	Seafood and Plant Proteins	Fatty Acids	Refined Grains	Sodium	Added Sugars	Saturated Fats
Max Score ^a^		100	5	5	5	5	10	10	5	5	10	10	10	10	10
All Outlets	2003–2004	54.75	2.86	3.17	3.30	2.10	1.80	6.20	5.00	4.38	4.16	5.56	4.97	5.49	5.82
	2005–2006	55.54	2.82	3.35	3.25	2.30	2.09	6.43	5.00	4.56	3.78	6.02	4.52	5.94	5.49
	2007–2008	56.69	3.03	4.06	3.26	2.30	2.07	6.31	5.00	4.44	3.97	6.13	4.35	5.84	5.68
	2009–2010	59.37	3.27	4.39	3.32	2.67	2.54	6.84	5.00	4.99	4.15	6.09	3.73	6.18	6.20
	2011–2012	60.18	3.00	4.01	3.36	2.98	2.87	6.44	5.00	5.00	4.66	6.19	4.15	6.28	6.23
	2013–2014	58.87	2.79	3.95	3.21	2.98	2.80	6.45	5.00	5.00	4.29	6.16	4.00	6.45	5.79
	2015–2016	58.74	2.88	4.19	3.35	3.11	2.97	6.01	5.00	5.00	4.12	6.41	3.72	6.85	5.14
	2017–2018	57.58	2.77	4.15	3.24	2.94	2.68	5.63	5.00	5.00	4.16	6.12	4.23	6.69	4.95
	*p* value	**0.03**	0.50	0.07	0.75	**0.00**	**0.00**	0.14	-	**0.02**	0.17	**0.03**	0.06	**0.00**	0.24
Stores	2003–2004	59.25	3.69	4.03	2.95	2.11	2.46	6.82	5.00	4.78	3.68	6.40	6.01	4.87	6.44
	2005–2006	60.75	3.71	4.44	2.86	2.25	2.87	7.11	5.00	4.91	3.24	7.01	5.92	6.06	5.36
	2007–2008	61.55	3.85	5.00	3.00	2.59	2.78	6.77	5.00	4.79	3.61	6.82	5.65	6.42	5.26
	2009–2010	63.52	4.03	5.00	3.16	2.85	3.34	7.32	5.00	5.00	3.80	6.79	4.68	6.74	5.82
	2011–2012	65.57	3.81	5.00	3.20	3.08	3.79	6.70	5.00	5.00	4.82	6.93	5.24	7.01	6.00
	2013–2014	63.93	3.63	5.00	3.04	3.19	3.58	6.69	5.00	5.00	4.22	7.01	5.18	6.39	6.01
	2015–2016	63.19	3.58	5.00	3.20	3.20	3.73	6.26	5.00	5.00	3.88	7.22	4.92	5.70	6.49
	2017–2018	62.27	3.52	5.00	3.05	3.10	3.50	5.85	5.00	5.00	3.95	7.02	5.59	5.50	6.19
	*p* value	0.07	0.27	0.24	0.09	**0.00**	**0.00**	**0.01**	-	0.90	0.10	**0.01**	0.28	**0.00**	0.33
Full-Service Restaurant	2003–2004	50.39	0.87	0.84	4.82	3.04	0.49	3.71	5.00	4.93	6.07	5.75	1.32	7.76	5.80
	2005–2006	49.32	0.69	0.70	4.72	3.62	0.49	3.80	5.00	5.00	5.80	5.15	0.49	5.47	8.41
	2007–2008	50.59	0.67	0.79	4.84	3.89	0.64	4.08	5.00	5.00	5.87	5.87	0.00	4.91	9.03
	2009–2010	49.78	0.78	0.83	4.31	3.32	0.62	4.68	5.00	5.00	5.29	5.85	0.71	5.11	8.29
	2011–2012	52.56	0.88	0.79	5.00	4.75	0.93	4.27	5.00	5.00	5.94	5.48	0.41	5.82	8.29
	2013–2014	53.99	0.67	0.95	4.69	4.70	1.05	3.63	5.00	5.00	6.79	5.92	0.34	6.46	8.79
	2015–2016	50.35	0.94	1.25	4.20	3.85	1.28	4.02	5.00	5.00	5.69	5.57	0.00	4.53	9.02
	2017–2018	50.70	0.47	0.53	4.61	4.25	0.82	3.83	5.00	5.00	6.21	5.63	0.00	5.11	9.25
	*p* value	0.34	0.08	0.29	0.34	0.06	**0.01**	0.96	-	0.99	0.46	0.97	0.99	**0.02**	0.80
Quick-Service Restaurant	2003–2004	38.68	0.63	0.47	3.65	1.49	0.42	5.37	5.00	2.11	4.62	1.90	2.86	6.44	3.70
	2005–2006	37.72	0.47	0.44	3.51	1.29	0.53	5.42	5.00	1.83	4.37	2.29	2.15	3.40	7.02
	2007–2008	38.62	0.56	0.60	3.18	1.51	0.48	5.52	5.00	2.20	4.39	3.14	2.25	3.24	6.55
	2009–2010	40.21	0.64	0.64	3.22	1.41	0.52	5.94	5.00	2.52	5.00	2.58	1.29	4.39	7.06
	2011–2012	41.76	0.73	0.86	3.26	2.03	0.82	6.83	5.00	2.48	4.05	3.33	2.03	3.72	6.62
	2013–2014	39.92	0.52	0.58	3.01	1.50	1.05	6.87	5.00	2.26	3.77	2.73	1.98	3.50	7.15
	2015–2016	40.73	0.64	0.80	3.23	2.30	0.88	5.90	5.00	2.64	4.23	2.84	1.42	3.17	7.69
	2017–2018	39.41	0.66	0.84	3.08	1.87	0.76	5.64	5.00	2.32	4.01	2.79	2.06	2.83	7.55
	*p* value	**0.01**	0.21	**0.02**	**0.01**	**0.05**	**0.02**	0.22	-	0.05	0.05	0.33	0.43	**0.01**	0.35
Schools	2003–2004	47.60	3.45	3.45	2.74	1.17	0.61	10	3.96	1.88	1.77	3.67	4.22	7.50	3.17
	2005–2006	48.82	3.53	3.57	2.59	0.99	0.69	10	4.09	1.28	2.66	4.82	3.57	7.19	3.85
	2007–2008	51.53	3.97	4.42	2.49	0.72	1.05	10	4.43	0.99	2.92	5.54	3.49	7.52	4.01
	2009–2010	49.88	4.57	5.0	1.98	1.09	1.09	10	3.83	1.24	1.80	3.43	3.49	7.80	4.56
	2011–2012	54.79	5.0	5.0	2.10	1.57	2.08	10	4.94	1.70	2.02	4.19	3.21	8.19	4.80
	2013–2014	61.92	5.0	5.0	2.71	1.65	5.61	10	4.22	1.69	2.82	5.61	3.54	8.32	5.76
	2015–2016	66.35	5.0	5.0	2.56	2.53	7.39	10	4.21	2.08	1.93	7.14	4.35	8.77	5.38
	2017–2018	64.99	5.0	5.0	2.17	1.43	7.24	10	3.57	2.52	2.10	5.79	5.76	8.43	6.0
	*p* value	**<0.001**	**0.001**	**0.01**	0.40	0.07	**0.006**	**-**	0.98	0.10	0.97	0.18	0.12	**0.002**	**<0.001**

Boldface indicates *p* < 0.05. ^a^ The maximum total or component score value represents a mix of foods aligned with the 2015–2020 Dietary Guidelines for Americans.

## Data Availability

Publicly available datasets were analyzed in this study. NHANES data can be found here: https://wwwn.cdc.gov/nchs/nhanes/Default.aspx (accessed on 6 August 2021).

## Supplementary Materials

The following are available online at https://www.mdpi.com/article/10.3390/nu13082717/s1, Table S1: *p*-values from Pairwise Comparisons Between Types of Food Outlets for HEI-2015 Total and Component Scores from Foods Consumed in the 2017–2018 NHANES.
