# Supplementary material for: Healthy Eating Index-2015 Scores Vary by Types of Food Outlets in the United States
Source: Nutrients. 2021 Aug 7;13(8):2717. doi: 10.3390/nu13082717 (PMC8398800; doi:10.3390/nu13082717)
Supplement: Supplementary file 1 [file nutrients-13-02717-s001.zip › nutrients-1322122-supplementary.pdf]

Supplemental Table S1. *p*-values from Pairwise Comparisons Between Types of Food Outlets for HEI-2015 Total and Component Scores from Foods Consumed in the 2017–2018 NHANES

Comparison	Total Score	Total Fruit	Whole Fruit	Total Vegetables	Greens and Beans	Whole Grains	Total Dairy	Total Protein	Seafood and Plant Protein	Fatty Acids	Refined Grains	Sodium	Saturated Fat	Added Sugar
School vs. Store	0.117	**<0.0001**	1.0	**0.001**	**0.0008**	**<0.0001**	**<0.0001**	**<0.0001**	**<0.0001**	**<0.0001**	0.595	**0.015**	0.296	**<0.0001**
School vs. Full-service restaurant	**<0.0001**	**<0.0001**	**<0.0001**	**<0.0001**	**<0.0001**	**<0.0001**	**<0.0001**	**<0.0001**	**<0.0001**	**<0.0001**	**<0.0001**	0.737	0.189	**0.031**
School vs. Quick-service restaurant	**<0.0001**	**<0.0001**	**<0.0001**	**0.0005**	**0.366**	**<0.0001**	**<0.0001**	**<0.0001**	0.694	**<0.0001**	**<0.0001**	**<0.0001**	**<0.0001**	**0.001**
Store vs. Full-service restaurant	**<0.0001**	**<0.0001**	**<0.0001**	**<0.0001**	**0.009**	**<0.0001**	**<0.0001**	.[a]	.	**<0.0001**	**0.002**	**<0.0001**	0.445	**<0.0001**
Store vs. Quick-service restaurant	**<0.0001**	**<0.0001**	**<0.0001**	0.802	**0.004**	**<0.0001**	0.403	.	**<0.0001**	0.857	**<0.0001**	**<0.0001**	**<0.0001**	**<0.0001**
Full-service restaurant vs. Quick-service restaurant	**<0.0001**	0.091	0.026	**<0.0001**	**0.001**	0.718	**<0.0001**	.	**<0.0001**	**<0.0001**	**<0.0001**	**<0.0001**	0.0004	0.0005

[a]: No significance reported for Seafood and Plant Protein for these comparisons as the same maximum score was achieved for these categories.
